# Finding local genome rearrangements

**DOI:** 10.1186/s13015-018-0127-2

**Published:** 2018-05-04

**Authors:** Pijus Simonaitis, Krister M. Swenson

**Affiliations:** 1 CNRS, LIRMM, Université Montpellier, 161 rue Ada, 34392 Montpellier, France; 2 Institut de Biologie Computationnelle (IBC), Montpellier, France

**Keywords:** Genome rearrangement, Double cut and join, Hi-C, Chromatin conformation, Maximum edge-disjoint cycle packing, NP-complete

## Abstract

**Background:**

The double cut and join (DCJ) model of genome rearrangement is well studied due to its mathematical simplicity and power to account for the many events that transform gene order. These studies have mostly been devoted to the understanding of minimum length scenarios transforming one genome into another. In this paper we search instead for rearrangement scenarios that minimize the number of rearrangements whose breakpoints are unlikely due to some biological criteria. One such criterion has recently become accessible due to the advent of the Hi-C experiment, facilitating the study of 3D spacial distance between breakpoint regions.

**Results:**

We establish a link between the minimum number of unlikely rearrangements required by a scenario and the problem of finding a maximum edge-disjoint cycle packing on a certain transformed version of the adjacency graph. This link leads to a 3/2-approximation as well as an exact integer linear programming formulation for our problem, which we prove to be NP-complete. We also present experimental results on fruit flies, showing that Hi-C data is informative when used as a criterion for rearrangements.

**Conclusions:**

A new variant of the weighted DCJ distance problem is addressed that ignores scenario length in its objective function. A solution to this problem provides a lower bound on the number of unlikely moves necessary when transforming one gene order into another. This lower bound aids in the study of rearrangement scenarios with respect to chromatin structure, and could eventually be used in the design of a fixed parameter algorithm with a more general objective function.

## Background

The problem of sorting genomes by a minimum number of biologically plausible rearrangements has been central to the theoretical comparative genomics community for roughly a quarter century. Traditionally, the likelihood of a rearrangement scenario has been based solely on the parsimony criterion. Unfortunately, a huge number of possible parsimonious scenarios between a pair of genomes exists [1–3]. This highlights the importance of methods that infer scenarios which conform to some extra biological constraints.

To this end we interest ourselves in data describing the 3D organization of chromatin, which is increasingly available due to the advent of an experiment called Hi-C [4, 5]. Indeed, the 3D spatial proximity of breakpoint regions have an important role in the formation [6, 7] and fixation [8] of genome rearrangements.

We have started development of methodology suitable for use with this type of constraint. Syntenic blocks of similar stretches of genomes are inferred, resulting in adjacencies that are candidate breakpoints for rearrangements. One can color these adjacencies for use with a cost function, where a DCJ acting on adjacencies having the same color is said to be *local*, and of zero cost, while a DCJ acting on adjacencies having different colors is *non-local*, and of cost one. In [9] we showed that the problem of finding—out of all parsimonious rearrangement scenarios—a scenario that minimizes the number of costly moves, the minimum local parsimonious scenario (MLPS) problem, is polynomial-time solvable. In this paper we disregard the parsimony criterion and instead focus solely on minimizing the number of costly moves required by a scenario, the minimum local scenario (MLS) problem.

In the "Minimum Local Scenario for circular genomes" section we treat a restricted case of MLS where only circular chromosomes are allowed. In the "Minimum Local Scenario for general genomes" section we show how general genomes can be capped in order to use the previously obtained results, establishing an exact formula for the number of costly moves in a minimum local scenario. This formula is based solely on the number of edges and the size of a maximum edge-disjoint cycle packing of the so-called *junction graph*, which is obtained by merging the vertices of the adjacency graph. We show that the MLS problem is NP-Complete, while admitting a 3/2-approximation (the "Complexity of MLS" section). We also implement an exact algorithm for MLS that is exponential in the number of colors but not in the number of genes. Despite the NP-hardness of MLS the exact algorithm is efficient enough to be applied to the comparison of *Drosophila melanogaster* and *Drosophila yakuba*.

We use the genomes of these fruit flies in the "Experiments" section to demonstrate the utility of MLS. We attribute colors to the adjacencies of *D. melanogaster* using *k*-medoid clustering, randomized clustering, and clustering based on the linear ordering of the adjacencies along the chromosomes. We observe a significant difference between the randomized clustering and the other two, while only a small difference between the *k*-medoid and linear clustering methods. We conclude that normalizing the Hi-C data before clustering is imperative, and that further study on both normalization and clustering would be beneficial for effective coloring of adjacencies.

Finally, a modification of MLS that attributes a non-zero cost to local moves could be of interest. Finding a minimum cost scenario in this case remains an open problem. In the "Towards a more general cost function" section, however, we provide an upper bound for the length of such a scenario, which is of interest in practice, as supported by our experimental results (the "Experiments" section).

## Definitions

### Genome and DCJ

A *genome* consists of *chromosomes* that are linear or circular molecules. Chromosomes are partitioned into uniquely labeled directed *syntenic blocks* separated by *breakpoint* regions.

**Fig. 1:**
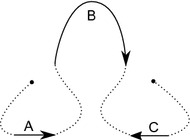
A genome consisting of a single linear chromosome partitioned into three syntenic blocks

A block has an orientation as indicated by an arrow, where the tail of the arrow represents the *tail extremity*, and the head of the arrow represents the *head extremity*. We can represent a genome by a set of *adjacencies* between extremities. Such a set for the genome from Fig. 1 is $\big \{\{A_{t}\}, \{A_{h}, B_{t}\}, \{B_{h},C_{h}\}, \{C_{t}\}\big \}$. An *adjacency* is either an unordered pair of the extremities that are adjacent on a chromosome, called *internal* adjacency, or a single extremity adjacent to one of the two ends of a linear chromosome, called an *external* adjacency.

#### **Definition 1**

(*Double cut and join*) A DCJ cuts one or two breakpoint regions and joins the resulting ends of the chromosomes back according to one of the following rules:

1. $$\{a,b\},\{c,d\}\rightarrow \{a,c\},\{b,d\}$$,
2. $$\{a,b\},\{c\}\rightarrow \{a,c\},\{b\}$$,
3. $$\{a,b\}\rightarrow \{a\},\{b\}$$,
4. $$\{a\},\{b\}\rightarrow \{a,b\}$$.

**Fig. 2:**
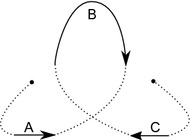
A genome obtained from the one presented in Fig. 1 via a DCJ $\{A_{h},B_{t}\},\{B_{h},C_{h}\}\rightarrow \{A_{h},B_{h}\},\{B_{t},C_{h}\}$ that inverts a block *B*

In Fig. 2 an example of a DCJ corresponding to an inversion of a syntenic block is provided.

### Renaming of block extremities

A DCJ cuts breakpoints and joins the resulting ends of the chromosomes while keeping the rest of the adjacencies in tact. The outcome of a DCJ only depends on whether the adjacencies it acts upon are internal or external. From Definition 1 we see that it does not matter if it acts on tail or head extremities, if the two adjacencies belong to different chromosomes, or if those chromosomes are linear or circular. This observation allows us to simplify the notation of genomes by renaming the block extremities. For example extremities of the genome from Fig. 1 can be renamed $(A_{h},B_{t},C_{h},B_{h},C_{t},A_{t})=(1,2,3,4,5,6)$ to obtain a set of adjacencies $\big \{\{1, 2\}, \{3,4\}, \{5\},\{6\}\big \}$. A DCJ scenario can be performed on these adjacencies. Once it is finished we can undo the renaming to obtain a new genome and a DCJ scenario leading to it. For example a move $\{1, 2\}, \{3,4\}\rightarrow \{1,4\},\{3,2\}$ results in a new set of adjacencies that, when renaming is undone, results in a genome $\big \{\{A_{h}, B_{h}\}, \{B_{t},C_{h}\}, \{C_{t}\},\{A_{t}\}\big \}$.

Given two genomes sharing the same $n+m$ syntenic blocks we can enumerate the extremities of these blocks to obtain the sets of adjacencies

$$\begin{aligned} A&= \big \{\{1,2\},\ldots ,\{2n-1,2n\}, \{2n+1\}, \ldots , \{2n+2m\}\big \},\\ B&= \big \{\{q_{1},q_{2}\},\ldots ,\{q_{2l-1},q_{2l}\}, \{q_{2l+1}\}, \ldots , \{q_{2n+2m}\}\big \}, \end{aligned}$$

where $\{1,2,\ldots ,2n+2m\}=\{q_{1},q_{2},\ldots , q_{2n+2m}\}$ and *A* has *n* internal and 2*m* external adjacencies (i.e. *m* linear chromosomes). A DCJ scenario transforming *A* into *B* implies a DCJ scenario transforming one genome into another. In what follows we will work with such sets of adjacencies, and without loss of generality we will call them *genomes*.

### Cost of a DCJ scenario

#### **Definition 2**

A coloring of the adjacencies of a genome *A* over a set of colors $\Delta$ is a function $col:A\rightarrow \Delta$ partitioning *A* into subsets of different colors.

A *coloring* is used to define the *cost* of a DCJ move. A move is *local* and of zero cost if it acts on adjacencies with equal colors, and it is *non-local* and of cost 1 otherwise. The cost of a sequence of DCJ moves, a DCJ *scenario*, is the sum of the costs of its constituent moves. For an adjacency $p\in A$ we use notation (*p*, *col*(*p*)) for a *colored adjacency*.

**Fig. 3:**
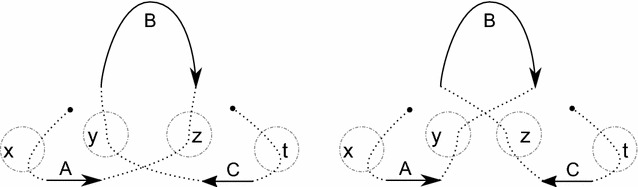
A DCJ move on the genome from Fig. 4 $\{A_{h},B_{t}\},\{B_{h},C_{h}\}\rightarrow \{A_{h},B_{h}\},\{B_{t},C_{h}\}$ that inverts a block *B* can have two different outcomes on a coloring

**Fig. 4:**
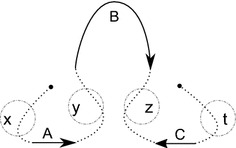
A set of colored adjacencies $\big \{(\{A_{t}\},x), (\{A_{h}, B_{t}\},y), (\{B_{h},C_{h}\},z), (\{C_{t}\},t)\big \}$ describes one possible coloring of the genome from Fig. 1 where every adjacency is attributed a unique color

A DCJ move might create two new colored adjacencies. If the adjacencies of colors *x* and *y* are broken by a DCJ and two new adjacencies are formed, then, under our model, one of them will be attributed color *x* and another color *y*. In Fig. 3 two possible outcomes of a DCJ move on the genome from Fig. 4 are presented. If an internal adjacency of color *x* is broken into two external adjacencies, then one of the adjacencies is attributed a color *x* and another is attributed any color *z*. The cost of such a move is 0 if and only if $z=x$.

#### **Definition 3**

The complete list of DCJs on colored adjacencies is:

1. $$(\{a,b\},x),(\{c,d\},y)\rightarrow (\{a,c\},x),(\{b,d\},y)$$ or $$(\{a,c\},y),(\{b,d\},x)$$,
2. $$(\{a,b\},x),(\{c\},y)\rightarrow (\{a,c\},x),(\{b\},y)$$ or $$(\{a,c\},y),(\{b\},x)$$,
3. $(\{a,b\},x)\rightarrow (\{a\},x),(\{b\},z)$ or $(\{a\},z),(\{b\},x)$ with any color *z*, and
4. $$(\{a\},x),(\{b\},y)\rightarrow (\{a,b\},x)$$ or $$(\{a,b\},y)$$.

The cost of a move is 0 if $x=y$ or $x=z$ and 1 otherwise.

In our previous work [9] we have treated the minimum local parsimonious scenario problem.

#### **Problem 1**

(*MLPS*) For genomes *A* and *B*, and a coloring of the adjacencies of *A*, find a minimum cost scenario among the DCJ scenarios of minimum length transforming *A* into *B*.

We have shown that MLPS is polynomial-time solvable. A real evolutionary scenario, however, might be non-parsimonious. In this paper we study the minimum local scenario problem which asks for potentially non-parsimonious scenarios.

#### **Problem 2**

(*MLS*) For genomes *A* and *B*, and a coloring of the adjacencies of *A*, find a minimum cost DCJ scenario transforming *A* into *B*.

### Adjacency and junction graphs

The *Adjacency* graph was introduced in [10] for the study of DCJ rearrangements. We introduce a transformation of the adjacency graph, called a *junction* graph, that incorporates the information on a coloring.

#### **Definition 4**

(*Adjacency graph*) For two genomes *A* and *B* the adjacency graph *AG*(*A*, *B*) is defined as an undirected bipartite multi-graph whose vertices are $A\cup B$, and there are exactly $|p\cap q|$ edges joining any $p \in A$ and $q\in B$.

#### **Definition 5**

(*Junction graph*) For two genomes *A*, *B* and a coloring *col* of *A* over $\Delta$ we define an undirected multi-graph $J(A,B,col)=(\Delta , E)$. For every internal adjacency $\{a,b\}\in B$ we add an edge (*x*, *y*) to *E* such that *x* and *y* are the colors of the adjacencies of *A* adjacent to $\{a,b\}$ in *AG*(*A*, *B*).

In what follows we will use letters *G*, *J* and *AG* when speaking about general graphs, junction graphs and adjacency graphs respectively.

#### **Definition 6**

(*2-break*) A 2-break is a transformation on a graph that replaces edges (*x*, *y*) and (*z*, *t*) with either (*x*, *z*) and (*y*, *t*), or (*x*, *t*) and (*y*, *z*).

#### Example 1

Consider the genomes from Figs. 1 and 2 with their block extremities renamed as described by the "Renaming of block extremities" section. They become

$$\begin{aligned} \begin{array}{r l} A = &{} \big \{\{1, 2\}, \{3,4\}, \{5\},\{6\}\big \}, \quad \text {and} \\ B = &{} \big \{\{1, 4\}, \{3,2\}, \{5\},\{6\}\big \}. \end{array} \end{aligned}$$

**Fig. 5:**
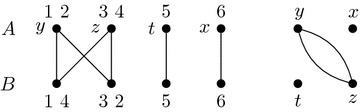
*AG*(*A*, *B*) on the left and *J*(*A*, *B*, *col*) on the right of the genomes *A* and *B* given in Example 1

**Fig. 6:**
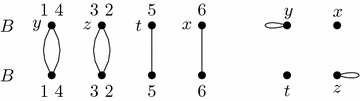
*AG*(*B*, *B*) on the left and $J(B,B,col')$ on the right of the genomes *A* and *B* given in Example 1. A transformation $J(A,B,col)\rightarrow J(B,B,col')$ is a 2-break $(y,z),(y,z)\rightarrow (y,y),(z,z)$

A coloring *col* of *A* is given in Fig. 4 where $col(\{1, 2\})=y$, $col(\{3, 4\})=z$, $col(\{5\})=t$, and $col(\{6\})=x$. We show *AG*(*A*, *B*) and *J*(*A*, *B*, *col*) in Fig. 5. A DCJ move $(\{1,2\},y),(\{3,4\},z)\rightarrow (\{1,4\},y),(\{3,2\},z)$ transforming *A* into *B* and coloring *col* into $col'$ transforms adjacency and junction graphs as presented in Fig. 6.

#### **Definition 7**

(*Eulerian graph*) If all the vertices of a graph *G* are of even degree, then *G* is said to be Eulerian.

## Minimum Local Scenario for circular genomes

In this section we treat the minimum local scenario problem in the restricted case where only circular chromosomes are allowed. In this case adjacencies will be called *pairs*. Given sets of pairs

$$\begin{aligned} A&= \big \{\{1,2\},\ldots ,\{2n-1,2n\}\big \},\\ B&= \big \{\{q_{1},q_{2}\},\ldots ,\{q_{2n-1},q_{2n}\}\big \}, \end{aligned}$$

with $\{1,2,\ldots ,2n\}=\{q_{1},q_{2},\ldots , q_{2n}\}$, our goal is to transform *A* into *B* using DCJ moves of the form $\{a,b\},\{c,d\}\rightarrow \{a,c\},\{b,d\}$.

In this case every vertex of an adjacency graph has degree two, all of its connected components are cycles and the junction graph is Eulerian. All connected components of *AG*(*B*, *B*) are cycles of length 2, thus at the end of a DCJ scenario transforming *A* into *B* we are left with a junction graph whose edges are all self-loops. We call such a graph *terminal*. For an Eulerian graph *G* we denote $\ell (G)$ as the minimum length of a 2-break scenario transforming *G* into a terminal graph.

### **Lemma 1**

*For sets of pairs A* *and B* *and a coloring col* *of A, the cost of a* minimum local scenario *transforming A* *into B is equal to* $\ell (J(A,B,col)).$

### *Proof*

We first transform any DCJ scenario into a 2-break scenario on *J*(*A*, *B*, *col*). From Example 1 it should be clear that for any DCJ move $A\rightarrow A'$, the transformation $J(A,B,col)\rightarrow J(A',B,col')$ is a 2-break. A DCJ move $A\rightarrow A'$ of cost zero can be disregarded since $J(A,B,col) = J(A',B,col')$. This means that a DCJ scenario of cost *w* transforming *A* (and its coloring *col*) into *B* (and its coloring $col_{B}$) provides us with a 2-break scenario of length at most *w* transforming *J*(*A*, *B*, *col*) into a terminal graph $J(B,B,col_{B})$. On the other hand for every 2-break $J\rightarrow J'$, a DCJ move $A\rightarrow A'$ can be found such that $J(A',B,col')=J'$. For any 2-break scenario of length *l* transforming *J*(*A*, *B*, *col*) into a terminal graph we obtain a DCJ scenario of length *l*, thus of cost at most *l*, transforming *A* (and its coloring *col*) into a genome *C* (and its coloring $col_{C}$) such that $J(C,B,col_{C})$ is terminal. This means that *C*’s pairs belonging to the same connected component of *AG*(*C*, *B*) are of the same color. A DCJ scenario transforming *C* into *B* and only acting on the pairs belonging to the same connected components of an adjacency graph can be easily found and such a scenario is of zero cost. This ensures that the scenario from *A* to *C*, and then from *C* to *B*, is a DCJ scenario transforming *A* into *B* of cost at most *l*. $\square$

### Linking 2-break scenarios and maximum edge-disjoint cycle packings

Using Lemma 1 we can shift our attention from a DCJ scenario on a set of pairs to a 2-break scenario on a junction graph *J*.

#### **Definition 8**

(*Maximum edge-disjoint cycle packing*) An MECP of a graph *G* is a largest set of edge-disjoint cycles in *G*. If *G* is Eulerian, then an MECP covers all of its edges.

For a graph $G=(V,E)$ we denote $e(G)=|E|$ and *c*(*G*) as the size of its MECP.

#### **Lemma 2**

*A 2-break on an Eulerian graph* *G can increase the size of its MECP by at most one.*

#### *Proof*

**Fig. 7:**
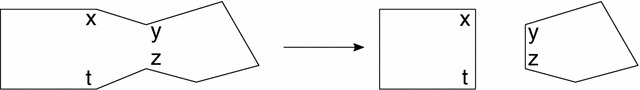
Given the two cycles of a cycle packing $C'$ of $G'$ containing edges (*x*, *t*) and (*y*, *z*) (on the right), we can apply a 2-break to get a cycle of *G* (on the left) containing edges (*x*, *y*) and (*z*, *t*)

Without loss of generality we can suppose that $G'$ is obtained from *G* via a 2-break replacing edges (*x*, *y*) and (*z*, *t*) by edges (*x*, *t*) and (*y*, *z*). We take an MECP of $G'$, call it $C'$, and construct a cycle packing *C* of *G* of size at least $|C'|-1$ to prove the claim. The set of edge-disjoint cycles of $C'$ that do not include edges (*x*, *t*) or (*y*, *z*) form a set of edge-disjoint cycles of *G*, so we include these cycles in *C*. If both newly added edges belong to the same cycle of $C'$, then we are done since in this case $|C|=|C'|-1$. Otherwise the edges of the two cycles containing edges (*x*, *t*) or (*y*, *z*) form a cycle in *G*, as depicted in Fig. 7, providing us with the last cycle for *C* implying $|C|=|C'|-1$. $\square$

#### **Lemma 3**

*For a graph G* *that is a cycle of length l there exists a 2-break scenario of length* $l-1$ *transforming it into a terminal graph.*

#### *Proof*

If $l=1$ then *G* is terminal. Otherwise there exists a 2-break that splits a cycle of length *l* into cycles of length 1 and $l-1$. We repeat this operation $l-1$ times to obtain a terminal graph. $\square$

#### **Theorem 1**

*For a junction graph* *J* *we have* $\ell (J)=e(J)-c(J).$

#### Proof

In our restricted case *J* is Eulerian, so its MECP covers all of its edges. We can transform the cycles of a given MECP one by one, thus obtaining a terminal graph at the end of a scenario. The length of such a scenario is $e(J)-c(J)$ using Lemma 3. On the other hand, the size of an MECP of a terminal graph is equal to *e*(*J*), and according to Lemma 2 we need at least $e(J)-c(J)$ 2-breaks to increase the size of an MECP from *c*(*J*) to *e*(*J*). $\square$

## Minimum Local Scenario for general genomes

Given two genomes

$$\begin{aligned} A&= \big \{\{1,2\},\dots ,\{2n-1,2n\}, \{2n+1\}, \ldots , \{2n+2m\}\big \},\\ B&= \big \{\{q_{1},q_{2}\},\dots ,\{q_{2l-1},q_{2l}\}, \{q_{2l+1}\}, \ldots , \{q_{2n+2m}\}\big \}, \end{aligned}$$

with $\{1,2,\ldots ,2n+2m\}=\{q_{1},q_{2},\ldots , q_{2n+2m}\}$, our goal is to transform *A* into *B* using DCJ moves defined in Definition 1.

### Genome capping

A genome can be extended into a set of pairs analyzed in the "Minimum Local Scenario for circular genomes" section by *capping*, which is the process of adding artificial gene extremities.

#### **Definition 9**

(*Genome extensions*) For a genome *A* we define its genome extension $\hat{A}$ to be a set of pairs of a form:

$$\begin{aligned}&\big \{\{1,2\},\ldots ,\{2n-1,2n\}, \{2n+1,\circ _{1}\}, \ldots , \{2n+2m,\circ _{2m}\},\\&\{\circ _{2m+1},\circ _{2m+2}\}, \ldots , \{\circ _{2m+2l-1},\circ _{2m+2l}\}\big \} \end{aligned}$$

where $l\in \mathbb {N}$ and $\{\circ _{1},\ldots , \circ _{2m+2l}\}= \{2n+2m+1, \ldots , 2n+4m+2l\}$. We define $A_{+}$ to be a set of all the possible genome extensions of *A*.

A pair $\{i,j\}$ where $i > 2n+2m$ and $j > 2n+2m$ will be called a *telomeric* pair. Internal adjacencies of a genome are present in its extension and external adjacencies are simply complemented by an artificial gene extremity. This means that adjacencies of a genome, and non-telomeric pairs of its extension, can be mapped one to one. A coloring *col* of *A* can be trivially extended to a coloring $\hat{col}$ of $\hat{A}\in A_{+}$ by keeping the same colors for the non-telomeric pairs, and by choosing any colors for the telomeric pairs.

For a DCJ move $A\rightarrow A'$ acting on two adjacencies of a genome there is an *induced* DCJ move $\hat{A}\rightarrow \hat{A}'$ of the same cost, where $\hat{A}'\in A'_{+}$ acting on the corresponding pairs of a genome extension. For example $(\{a\},x),(\{b\},y)\rightarrow (\{a,b\},x)$ induces $(\{a,\circ _{i}\},x),(\{b,\circ _{j}\},y)\rightarrow (\{a,b\},x),(\{\circ _{i},\circ _{j}\}, y)$ and $(\{a,b\},x),(\{c\},y)\rightarrow (\{a,c\},x),(\{b\},y)$ induces $(\{a,b\},x),(\{c,\circ _{i}\},y)\rightarrow (\{a,c\},x),(\{b,\circ _{i}\},y)$. A DCJ move of the form $(\{a,b\},x)\rightarrow (\{a\},x),(\{b\},z)$ (i.e. acting on a single adjacency) is different, as in this case we need a telomeric pair of color *z* to be present in a genome extension. For example $(\{a,b\},x)\rightarrow (\{a\},x),(\{b\},z)$ induces $(\{a,b\},x), (\{\circ _{i},\circ _{j}\},z)\rightarrow (\{a,\circ _i\},x),(\{b,\circ _j\},z)$ on a genome extension including $(\{\circ _{i},\circ _{j}\},z)$.

#### **Lemma 4**

*For a DCJ scenario transforming genome* *A into B and a coloring of* *A, there exists genome extensions* $\hat{A}\in A_{+}$ *and* $\hat{B}\in B_{+},$ *as well as a scenario of the same cost transforming* $\hat{A}$ *into* $\hat{B}.$

#### *Proof*

In the DCJ scenario transforming *A* into *B*, let *p* be the number of DCJ moves acting on a single adjacency. These are of a form $\{a,b\}\rightarrow \{a\}, \{b\}$. We take a genome extension $\hat{A}\in A_{+}$ with *p* telomeric pairs. Every DCJ move $(\{a,b\},x)\rightarrow (\{a\},x),(\{b\},z)$ will induce a move acting on a different telomeric pair of a genome extension and its color will be the color *z* required by the DCJ move on the non-extended genome. In this way every DCJ move on a genome will induce a move on a genome extension, and after a scenario of cost *w* we will end up with $\hat{B}$, an extension of genome *B*. $\square$

#### **Lemma 5**

*For a DCJ scenario transforming* $\hat{A}\in A_{+}$* into* $\hat{B}\in B_{+}$* and a coloring of* *A there exists a DCJ scenario of the same cost or smaller transforming A* into *B*.

#### *Proof*

We start with a pair $(A,\hat{A})$ and apply a scenario transforming $\hat{A}$ into $\hat{B}$ step by step, updating *A* along the way accordingly. After the first *k* moves of a scenario whose cost is $w_{k}$ we get a pair $(A^{k},\hat{A}^{k})$ with $\hat{A}^{k}\in A^{k}_{+}$ such that there is a scenario of cost at most $w_{k}$ transforming *A* into $A^{k}$. A pair $(A^{k+1},\hat{A}^{k+1})$ is constructed as follows. The $k+1$st move of a scenario is $\hat{A}^{k}\rightarrow \hat{A}^{k+1}$.

If $\hat{A}^{k+1}\in A^{k}_{+}$, then the next pair is $(A^{k},\hat{A}^{k+1})$. If $\hat{A}^{k+1}\notin A^{k}_{+}$, then for a DCJ $(\{a_{1},a_{2}\},x),(\{a_{3},a_{4}\},y)\rightarrow (\{a_{1},a_{3}\},x),(\{a_{2},a_{4}\},y)$ transforming $\hat{A}^{k}$ into $\hat{A}^{k+1}$, at least one of the four involved pairs must contain two gene extremities. This observation allows us to find a genome *C* such that $\hat{A}^{k+1}\in C_{+}$ and there is a DCJ move $A^{k}\rightarrow C$ of the same cost as $\hat{A}^{k}\rightarrow \hat{A}^{k+1}$. Therefore the next pair is $(C, \hat{A}^{k+1})$. There are numerous cases for *C*, but they are all trivial to analyze. For example, if $a_{1}$ and $a_{2}$ are both gene extremities and $\{a_{3},a_{4}\}$ is a telomeric pair then a DCJ $(\{a_{1},a_{2}\},x)\rightarrow (\{a_{1}\},x),(\{a_{2}\},y)$ transforms $A^{k}$ into such a *C*.

Now $\hat{A}^{k+1}\in A^{k+1}_{+}$ and the scenario transforming *A* into $A^{k+1}$ is of cost at most $w_{k+1}$. We continue until we obtain $(B,\hat{B})$ with a scenario transforming *A* into *B* of cost at most *w*. $\square$

Define an *Eulerian extension* of a graph to be an Eulerian graph obtained from the initial graph by adding some edges. By construction $J(\hat{A},\hat{B},\hat{col})$ is an Eulerian extension of *J*(*A*, *B*, *col*). We close this section by relating Eulerian extensions of *J*(*A*, *B*, *col*) to the junction graphs of genome extensions.

#### **Lemma 6**

*For every Eulerian extension* $J'$* of* $J=J(A,B,col)$* there exists genome extensions* $\hat{A}\in A_{+}$* and* $\hat{B}\in B_{+}$* such that* $J(\hat{A},\hat{B},\hat{col})$* and* $J'$* have exactly the same non-loop edges. We say that such graphs are* *non-loop equal*.

#### *Proof*

**Fig. 8:**
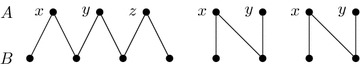
The adjacency graph *AG*(*A*, *B*) consisting of three paths. Adjacencies of *A* are colored using colors *x*, *y*, *z* as indicated

**Fig. 9:**
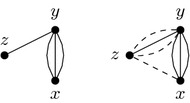
The junction graph *J*(*A*, *B*, *col*) on the left and one possible Eulerian extension $J'$ on the right

We will augment *AG*(*A*, *B*) with new adjacencies to obtain intermediate versions of adjacency graph $AG'$ before finally arriving at an $AG' = AG(\hat{A},\hat{B})$. Our running example will use the graphs shown in Figs. 8 and 9.

**Fig. 10:**
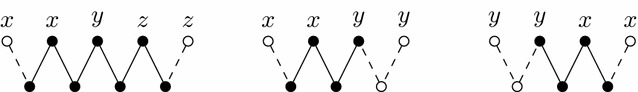
$AG'$ after the paths of *AG*(*A*, *B*) are extended. *AG*(*A*, *B*) is a subgraph of $AG'$. Newly added vertices end edges are white and dashed respectively

**Fig. 11:**
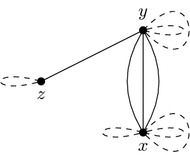
The junction graph of $AG'$ is loop equal to *J*

$AG'$ has exactly the cycles of *AG*(*A*, *B*). The paths of *AG*(*A*, *B*), however, appear in $AG'$ in a modified form. To each path we add new vertices at its endpoints, copying the endpoints’ colors (see Fig. 10 for an example). The junction graph of $AG'$ is now non-loop equal to *J*. In our example *AG*(*A*, *B*) has no cycles and its three paths imply paths for $AG'$ as shown in Fig. 10. The junction graph of $AG'$ is given in Fig. 11.

**Fig. 12:**
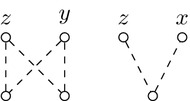
The extension to $AG'$ yielding $J'- J$ as its junction graph

For graphs $G'=(V,E\cup E')$ and $G=(V,E)$ we denote $G'-G=(V,E')$. Take an Eulerian subgraph *H* of $J'-J$ such that $F=(J'-J)-H$ is a forest. For *H* we add a union of cycles to $AG'$ such that *H* is the junction graph of these cycles. *F* can be partitioned into paths joining the vertices of odd degree, and for each of these paths we add to $AG'$ a path with adjacencies of the corresponding colors. In our example, *H* is a cycle (*z*, *y*, *z*) and *F* has a single path (*z*, *x*). These add a cycle and a path to $AG'$, shown in Fig. 12.

**Fig. 13:**
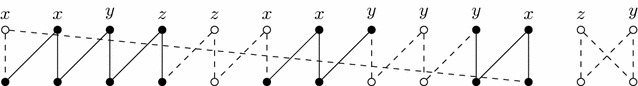
$AG'$ after merging the endpoints of the paths

**Fig. 14:**
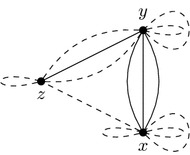
The junction graph of $AG'$, which is non-loop equal to $J'$

Now transform every path of $AG'$ into a cycle in the following way. Each path of $AG'$ that has endpoints of the same color is now transformed into a cycle by merging those endpoints into a single vertex of degree two. We are left with paths having endpoints of different colors. Consider one such path with an endpoint of color *x*. Merge this vertex with an endpoint of color *x* from another path. Such a path will always exist since every vertex in the Eulerian extension $J'$ has an even degree, implying that there is an even number of paths in $AG'$ having this vertex colored *x* as an endpoint. Continue this procedure until no paths are left. One possible outcome of such an operation is given in Fig. 13. The junction graph of $AG'$ is now non-loop equal to $J'$ as it can be seen in Fig. 14.

It is easy to reconstruct $\hat{B}\in B_{+}$, $\hat{A}\in A_{+}$ and its coloring $\hat{col}$ such that $AG'=AG(\hat{A},\hat{B},\hat{col})$, which guarantees that $J(\hat{A},\hat{B},\hat{col})$ is non-loop equal to $J'$. $\square$

### A closed formula for minimum local scenario

#### **Theorem 2**

*The cost* *w* *of a* minimum local scenario *transforming genome* *A* *into* *B is* $e(J)-c(J)$ *where* $J=J(A,B,col).$

#### Proof

For a cycle packing *C* of *J* of cardinality *c*(*J*), define an Eulerian extension $J'$ by duplicating every edge of *J* not belonging to *C*. Denote the number of such edges by *k*. A union of *C*, and the *k* cycles of length 2 created by the added edges, will be a cycle packing $C'$ of $J'$. Using Theorem 1 we obtain

$$\begin{aligned} \ell (J')&=e(J')-c(J') \le e(J')-|C'|=e(J)+k-c(J)-k=e(J)-c(J), \end{aligned}$$

where $\ell (J')$ is the minimum length of a 2-break scenario transforming $J'$ into a terminal graph. Using Lemma 6 we construct sets of pairs $\hat{A}\in A_{+}$ and $\hat{B}\in B_{+}$ such that $J(\hat{A},\hat{B},\hat{col})$ is non-loop equal to $J'$. Using Lemma 1 we construct a DCJ scenario of cost at most $\ell (J')$ transforming $\hat{A}$ into $\hat{B}$, from which we can construct a DCJ scenario of cost at most $\ell (J')$ transforming *A* into *B* while using Lemma 5. This implies that $w\le \ell (J')\le e(J)-c(J)$.

For a DCJ scenario of cost *w* transforming *A* into *B* we use Lemma 4 to construct the sets of pairs $\hat{A}\in A_{+}$ and $\hat{B}\in B_{+}$, and a scenario of cost *w* transforming $\hat{A}$ into $\hat{B}$. This leads to a 2-break scenario transforming $J'=J(\hat{A},\hat{B},\hat{col})$ into a terminal graph of length at most *w* using Lemma 1. Theorem 1 ensures an existence of a cycle packing $C'$ of $J'$ such that $w\ge e(J')-|C'|$. Define *C* to be the union of the cycles in $C'$ consisting entirely of the edges of $J=J(A,B,col_{A})$. Counting edges and cycles gives

$$\begin{aligned} w\ge e(J')-|C'|=e(J)-|C|+e(J')-e(J)-|C'\setminus C|. \end{aligned}$$

Due to the construction of *C* every cycle in $C'\setminus C$ admits at least one edge from $J'$ not belonging to *J*, and thus $e(J')-e(J)\ge |C'\setminus C|$. So we have inequality $w\ge e(J)-|C|\ge e(J)-c(J)$. $\square$

Constructions in lemmas 1, 4, 5 and 6 used in Theorem 2 are all polynomial. The next section shows that computing *c*(*J*) is at the heart of the complexity of the minimum local scenario.

## Complexity of MLS

### NP-completeness of MLS

#### **Theorem 3**

*The decision version of* minimum local scenario *is NP-complete.*

#### Proof

The decision version of MLS is clearly in NP. We reduce the decision version of MECP on Eulerian graphs, which is NP-hard [11] (and APX-hard [12]), to MLS. Without loss of generality, take an instance $G=(V,E)$ and a bound *k* of MECP, where *G* is Eulerian and connected. We will construct genomes *A*, *B* and a coloring *col* such that $J(A,B,col)=G$. Consider an Eulerian tour $u_{1},u_{2},\dots ,u_{n},u_{1}$ of *G* and set

$$\begin{aligned} A&= \big \{\{1,2\},\{3,4\},\ldots ,\{2n-1,2n\}\big \},\, \text {and} \\ B&= \big \{\{2,3\},\{4,5\},\ldots ,\{2n,1\}\big \}. \end{aligned}$$

The vertices of *V* are the colors of *col* such that $col\big (\{2i-1,2i\}\big )=u_{i}$ for all $i\in \{1,\ldots ,n\}$. By construction $J(A,B,col) = G$. Theorem 2 says that an optimal solution to MLS of cost *w* implies the existence of a cycle packing of size $e(G)-w$. Thus there is an MECP of size *k* if and only if $e(G)-w \ge k$. $\square$

### A 3/2-approximation for MLS

For a cycle packing *C* of a graph *G*, denote the number of its length-one and length-two cycles by $c_{1}(C)$ and $c_{2}(C)$ respectively, and the number of longer cycles by $c_{+}(C)$. Denote the number of *G*’s edges and length-one cycles by *e*(*G*) and $c_{1}(G)$ respectively. Finally, denote by $c_{2}(G)$ the maximum of $c_{2}(C)$ among all of the cycle packings of *G*.

#### **Lemma 7**

*For every Eulerian graph G*

$$\begin{aligned} \ell (G)\ge \frac{2}{3}e(G)-\frac{1}{3}c_{2}(G)-\frac{2}{3}c_{1}(G). \end{aligned}$$

#### *Proof*

$\ell (G)=e(G)-c(G)$ using Theorem 1. For a maximum edge-disjoint cycle packing *C* of *G* we have $c(J)=|C|=c_{1}(C)+c_{2}(C)+c_{+}(C)$ and $e(G)\ge c_{1}(C)+2c_{2}(C)+3c_{+}(C)$, which implies

$$\begin{aligned}&\ell (G)-\frac{2}{3}e(G)=\frac{1}{3}e(G)-|C|\ge -\frac{1}{3}c_{2}(C)-\frac{2}{3}c_{1}(C),~\text {so}\\&\ell (G)\ge \frac{2}{3}e(G)-\frac{1}{3}c_{2}(C)-\frac{2}{3}c_{1}(C) \quad \ge \frac{2}{3}e(G)-\frac{1}{3}c_{2}(G)-\frac{2}{3}c_{1}(G). \end{aligned}$$

$$\square$$

#### **Theorem 4**

*For genomes* *A,* *B and a coloring* *col of* *A, the cost* $w_{\mathrm{MLS}}$ *of a MLS transforming* *A* *into* *B* *respects*

$$\begin{aligned} w_{\mathrm{MLS}} \ge \frac{2}{3}e(J)-\frac{1}{3}c_{2}(J)-\frac{2}{3}c_{1}(J), \text {where } \,J=J(A,B,col). \end{aligned}$$

#### Proof

Take an MECP *C* of *J*. It covers an Eulerian subgraph $J'$ of *J*. Using Theorem 2 we have $w_{{MLS}}=e(J)-|C|$, and by counting edges and using Theorem 1 we obtain

$$\begin{aligned} w_{\mathrm{MLS}} =e(J)-|C|= e(J')-|C|+e(J)-e(J')=\ell (J')+e(J)-e(J'), \end{aligned}$$

from which using Lemma 7 and a simple counting argument we get

$$\begin{aligned} w_{\mathrm{MLS}} \ge \frac{2}{3}e(J')-\frac{1}{3}c_{2}(J')-\frac{2}{3}c_{1}(J')+e(J)-e(J')\ge \\ \frac{2}{3}e(J)-\frac{1}{3}c_{2}(J)-\frac{2}{3}c_{1}(J). \end{aligned}$$

$$\square$$

In Theorem 2 we have shown how a cycle packing *C* of $J=J(A,B,col)$ gives a DCJ scenario of cost $w\le e(J)-|C|$ transforming *A* into *B*. For a cycle packing *C* consisting of $c_{2}(J)$ pairwise edge-disjoint cycles of length two and $c_{1}(J)$ loops, there is a scenario of cost $w\le e(J)-c_{2}(J)-c_{1}(J)=w'$. Using Theorem 4 we have

$$\begin{aligned} w_{\mathrm{MLS}}\ge \frac{2}{3}e(J)-\frac{1}{3}c_{2}(J)-\frac{2}{3}c_{1}(J)=\frac{2}{3}(w'+\frac{1}{2}c_{2}(J)), \end{aligned}$$

yielding the approximation ratio

$$\begin{aligned} \alpha =\frac{w}{w_{\mathrm{MLS}}}\le \frac{3}{2}\frac{w}{w'+\frac{1}{2}c_{2}(J)}\le \frac{3}{2}. \end{aligned}$$

### An exact algorithm for MLS

Consider a junction graph *J* with $c_{1}(J)$ loops and $c_{2}(J)$ length two cycles. The observation that there exists an MECP of *J* that includes all of these cycles allows us to simplify the problem by removing them from *J*. This leaves us with a simple graph $\bar{J}$ such that the cost of MLS is equal to $e(J)-c_{1}(J)-c_{2}(J)-c(\bar{J})$. A straightforward way to compute $c(\bar{J})$ is to take the set *S* of all of $\bar{J}$’s simple cycles and solve the maximum set packing problem on their sets of edges formulated as an integer linear program:

$$\begin{aligned} \text {Maximize }&\sum _{c\in S} x_{c}\\ \text {Subject to }&\sum _{c:e\in c}x_{c}\le 1\text { for each edge } e \text { of } \bar{J}\\ \text {and }&x_{c}\in \{0,1\} \text { for every simple cycle } c\in S. \end{aligned}$$

The number of simple cycles might be exponential in the number of colors and not the number of syntenic blocks. We see in the "Experiments" section that our algorithm solves MLS on instances between *Drosophila melanogaster* and *Drosophila yakuba*.

## Towards a more general cost function

Our work opens the door to the development of a more general model for genome rearrangements with positional constraints, where local moves are attributed nonzero cost. In such a model the costs of local and non-local moves would be respectively $\omega _L$ and $\omega _N$ with $0<\omega _L<\omega _N$. For any DCJ scenario $\rho$ we will denote $\omega (\rho )$, $N(\rho )$ and $L(\rho )$ as its cost, its number of non-local, and local moves respectively. We categorize the different DCJ problems based on the cost pair $(\omega _L, \omega _N)$ with $0\le \omega _L\le \omega _N$, where we look for a $\rho$ that minimizes the cost function $\omega (\rho ) = \omega _L L(\rho ) + \omega _N N(\rho )$:

- (0, 1) is the minimum local scenario problem,
- (1, 1) is the traditional double cut and join problem,
- $(\omega _L, \omega _N)$ with $\frac{\omega _L}{\omega _N-\omega _L}>n$, where *n* is the number of adjacencies, is the minimum local parsimonious scenario problem,
- $(\omega _L, \omega _N)$ with $0<\omega _L<\omega _N$ is the problem that we consider in this section.

It is clear that for positive *k* the cost pairs $(\omega _L,\omega _N)$ and $(k\omega _L,k\omega _N)$ define the same minimum scenarios, so for $0<\omega _L<\omega _N$ it suffices to treat the normalized pair $(1,1+\alpha )$ with a positive $\alpha$. For a scenario $\rho$ we denote $\delta (\rho ) = N(\rho )+L(\rho )-d_{DCJ}$ as the difference of its length and the length of a parsimonious DCJ scenario. If $\delta$ were small we would have an algorithmic tool in the search for genomic distances. For $(\omega _L,\omega _N)=(1,1+\alpha )$, we have

$$\begin{aligned} \omega (\rho ) = L(\rho )+N(\rho )+N(\rho )\alpha =\delta (\rho )+d_{DCJ}+N(\rho )\alpha ,~ \end{aligned}$$

By $d_{MLPS}$ and $d_{MLS}$ we denote the numbers of non-local moves in minimum local parsimonious scenario and minimum local scenario respectively. For a scenario $\rho ^*$ minimizing the cost $L(\rho )+(1+\alpha )N(\rho )$ we have $d_{DCJ}+d_{MLPS}\alpha \ge \omega (\rho *)$, as $d_{DCJ}+d_{MLPS}\alpha$ is the cost of a MLPS. Subtracting $d_{DCJ}$ we obtain $d_{MLPS}\alpha \ge \delta (\rho *)+N(\rho ^*)\alpha$. By definition $N(\rho ^*)\ge d_{MLS}$, and so we obtain

$$\begin{aligned} (d_{MLPS}-d_{MLS})\alpha \ge \delta (\rho ^*). \end{aligned}$$

In general $d_{MLPS}-d_{MLS}$ might be large, however we have shown in [13] that at least for *D. melanogaster* and *D. yakuba* it is small in practice. This means that the problem of finding a scenario of minimum cost among those with a small $\delta$, for example $\delta =1$, might be of practical interest.

## Experiments

Our theoretical work is based on a coloring of adjacencies where rearrangements are considered to be more likely when acting on those of the same color, and where the colors of the adjacencies are preserved across large evolutionary distances. Although there are many factors that may effect the likelihood of a rearrangement, in this section we focus on a coloring that partitions a genome into local 3D regions using Hi-C data. This idea is supported by the hypothesis that rearrangements are more likely to occur between breakpoints in close spatial proximity [6, 7], and an observation that syntenic blocks distant in the 1D sense in human, but adjacent in mouse, were observed in close 3D proximity in the human more often than expected [8].

We use Hi-C data as a similarity function for pairs of adjacencies. We propose a simple weight function of a coloring based on this similarity, and an algorithm k-medoidsthat provides colorings maximizing the weight. Unfortunately it is not clear exactly how Hi-C values are linked to 3D distance in the nucleus, thus there is no definitive way to know how well our clusters capture the 3D structure of a genome.

We compare these colorings to two other clustering algorithms: linear, which respects the 1D structure of the chromosomes, and random, which attributes colors to adjacencies at random. We report results in the "Clustering algorithms", "Colorings inferred from the adjacency graph" and "Divergence from linearity" sections.

All results of this section are possible due to the fact that, despite the NP-hardness of the minimum local scenario problem, we find that it can be computed exactly (using our algorithm from the "An exact algorithm for MLS" section) for all of the colorings obtained for *D. melanogaster* and *D. yakuba*.

### Hi-C data and normalization

A Hi-C experiment is conducted on a population of cells, thereby providing a rough estimate of the number of cells in which a pair of genomic loci were found to be in close 3D proximity. Hi-C estimates of the number of cells in which a pair of genomic loci were found to be in close 3D proximity are organized into matrices of contacts within fixed-sized windows. Due to the nature of Hi-C contacts, which decrease dramatically with respect to chromosomal distance (it roughly follows a power law), we applied the normalization done by Lieberman-Aiden et al. (see the appendix of [4]) to the matrices published in [5]. For intra-chromosomal matrices, this normalization ensures that rearrangements with distant breakpoints (in the *1D* genetic coordinate sense) have increased relative importance to the close ones (in 1D). Specifically, a normalized intra-chromosomal heatmap entry $INTRA_{ij}$ gets the value

$$\begin{aligned} INTRA_{ij} = H_{ij} / averageAtDist(|i-j|), \end{aligned}$$

where *averageAtDist*(*d*) is the expected Hi-C value of two loci separated by distance *d* (in the 1D sense) over all chromosomes. A normalized inter-chromosomal Hi-C value is

$$\begin{aligned} INTER_{ij} = H_{ij} / \Big (\frac{interaction_i*interaction_j}{interaction_{all}}\Big ), \end{aligned}$$

where $interaction_x$ is the sum of all Hi-C values for locus *x*, and $interaction_{all}$ is the sum of all Hi-C values (intra- *and* inter-chromosomal). This inter-chromosomal normalization accentuates the importance of values that come from loci with a bias towards fewer contacts, while diminishing the importance of values that come from loci with a bias towards a large number of contacts.

We use both non-normalized and normalized Hi-C data as similarity functions for pairs of adjacencies. These similarity functions are used to define the weight of a coloring.

### Clustering algorithms

In what follows the terms *coloring* and *clustering* will be used interchangeably. We perform our experiments on the genomes of *D. melanogaster* and *D. yakuba* which were partitioned into 64 syntenic blocks using *Orthocluster* tool [14]. The DCJ distance between the two genomes is 51. *D. melanogaster* is used as genome *A* with a coloring that we obtain from the Hi-C data published in [5]. No Hi-C for *D. yakuba* is publicly available at the time of writing.

The first of the clustering methods we used is clustering around medoids, which was chosen for its simplicity and speed [15]. A *medoid* of a cluster is an element that maximizes the sum of the similarities to the rest of a cluster. This sum is the cluster’s *weight*, and when summed over all the clusters it provides us with a *clustering weight*. We use this algorithm with Hi-C data as a similarity function for the pairs of adjacencies.

The k-medoids algorithm starts with *k* randomly initialized centroids. The rest of the elements are then associated to the centroids that are most similar to them. The medoids of the obtained clusters are then computed and they become the new centroids around which the elements will be clustered. We continue this procedure until the clustering weight stops increasing.

The linear algorithm respects the 1D structure of the 6 chromosome arms of *D. melanogaster*. For a given $k\ge 6$ we choose $k-6$ syntenic blocks at random and cut the chromosomes at these blocks. In this way we obtain *k* segments of the chromosomes, each of which has at least one adjacency. We then attribute a distinct color to each segment and assign to each adjacency the color of its segment.

The random algorithm attributes colors to the adjacencies uniformly at random while ensuring that for every color there is at least one adjacency of that color.

**Fig. 15:**
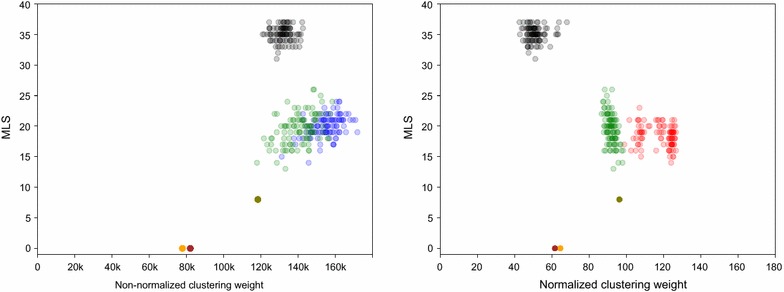
Comparison of MLS and clustering weights for random (black points), k-medoids (non-normalized blue and normalized red points) and linear (green points) clusterings for $k=15$ clusters. minimum linear  (olive point) is a manually constructed linear clustering with the smallest possible MLS (defined in the "Colorings inferred from the adjacency graph" section). optimal (orange and brown) points are clusterings with 0 MLS (defined in the "Divergence from linearity" section). The mean MLS value is 35 for random, 19.5 for linear, 20.0 for blue k-medoids points, and 18.4 for red k-medoids points

Clustering weight is well defined for any clustering. Non-normalized and normalized Hi-C data provides two similarity functions for the pairs of adjacencies and thus two different clustering weights: non-normalized and normalized. In Fig. 15 we compare MLS to clustering weights for the number of colors $k=15$. We generate 100 random clusterings (black) and 100 linear clusterings (green) and compute their normalized and non-normalized clustering weights and MLS. We generate 100 k-medoids clusterings using non-normalized Hi-C (blue) and 100 k-medoids clusterings using normalized Hi-C (red). The meaning of olive, brown and orange outliers in Fig. 15 will be explained in the "Colorings inferred from the adjacency graph" and "Divergence from linearity" sections.

Both linear and k-medoids have significantly lower MLS than random clusterings, however the MLS is very similar for linear and both k-medoids clusterings, with average cost being close to 19. Separation between the clustering weights of linear, random and k-medoids is more pronounced for normalized Hi-C.

### Colorings inferred from the adjacency graph

The k-medoids, linearand random algorithms color the adjacencies of a single genome using Hi-C data or 1D structure. However if we use the adjacencies of both genomes we can construct colorings with a much lower MLS cost. For example, if we chose a coloring for which the connected components of an adjacency graph are monochromatic, then the junction graph is terminal and MLS is equal to 0. The adjacency graph of *D. melanogaster* and *D. yakuba* has 19 connected components. This means that for $k\le 19$ there exists a coloring with MLS equal to 0. We call such coloring optimal.

Another extreme case is a linear coloring minimizing MLS. When every chromosome is assigned its own color MLS is equal to 8. By increasing the number of colors for a linear clustering we can only increase its MLS, thus a lower MLS bound for linear clustering is 8. This lower bound was not observed when running linear, however using the adjacency graph of *D. melanogaster* and *D. yakuba* we have manually constructed colorings of up to 19 colors with MLS equal to 8. We call these colorings minimum linear.

### Divergence from linearity

Clusters in optimal and k-medoids clusterings mostly contain adjacencies from only one or two chromosomes. Moreover, those adjacencies are mostly contiguous in the 1D sense on the chromosomes. We define *divergence from linearity* in order to quantify *non-linearity* of these colorings, and to see if it is related to MLS.

#### **Definition 10**

(*Divergence from linearity*) Consider a set *S* of adjacencies colored with color *x*. Partition it into nonempty subsets $S_{1}, S_{2}\ldots , S_{l}$ of adjacencies belonging to the chromosomes $chr_{1}, chr_{2}\ldots , chr_{l}$. Sort these subsets according to the 1D structure of the chromosomes. Say that $S_{i}$ has a *gap* if there is an adjacency *p* followed by *q* in $S_{i}$, but in $chr_{i}$ there is some adjacency *r* in between *p* and *q*. The *divergence from linearity* of *S* is the total number of gaps in *S* plus $l-1$. Sum these values for all the colors to obtain the *divergence from linearity* for a coloring.

The divergence from linearity of the linear coloring is 0. We use a greedy algorithm to find optimal colorings minimizing and maximizing the divergence from linearity.

**Fig. 16:**
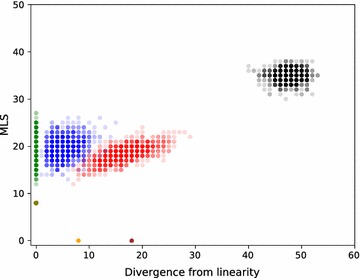
A comparison of MLS and divergence from linearity for the number of clusters $k=15$. We generated 1000 random (black), linear (green), and k-medoids clusterings using non-normalized Hi-C (blue) and k-medoids clusterings using normalized Hi-C (red). minimum linear (olive), optimal minimizing divergence from linearity (orange), and optimal maximizing divergence from linearity (brown) are also plotted. Mean divergence from linearity for blue k-medoids points is 5.3 and 16.5 for red k-medoids points

In Fig. 16 we compare MLS to the divergence from linearity for $k=15$ colors. We observe that k-medoids clusters using non-normalized Hi-C are more linear than those using normalized Hi-C, with their mean values being 5.3 and 16.5 respectively. This was expected due to the nature of normalization, which accentuates the importance of the Hi-C values of the adjacencies that are distant in 1D sense or coming from different chromosomes.

We see that the linear and k-medoids clusterings for non-normalized Hi-C are almost the same due to the low divergence from linearity of the latter. This explains why they have almost the same non-normalized clustering weight, as can be seen in Fig. 15. optimal and minimum linear provides the bounds for MLS and divergence from linearity.

**Fig. 17:**
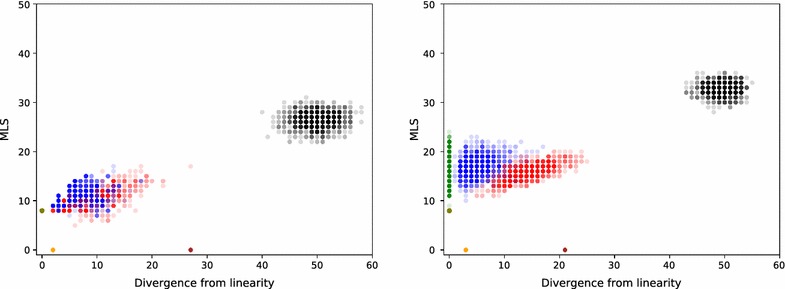
A comparison of MLS and divergence from linearity for the number of clusters $k=6$ on the left and $k=12$ on the right. The clusterings and their colors in the plot are exactly as in Fig. 16. For $k=6$ there exists a single linear clustering where the 6 chromosomes are assigned unique colors, and it coincides with the minimum linear clustering

**Fig. 18:**
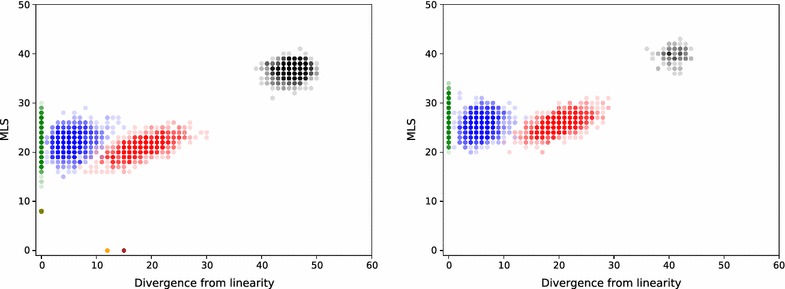
Comparison of MLS and divergence from linearity for the number of clusters $k=18$ on the left and $k=24$ on the right. For $k=18$ the clusterings and their colors in the plot are exactly as in Fig. 16. For $k=24$ there are 100 random (black points) clusterings as opposed to 1000 of Fig. 16. optimal clusterings do not exist for $k>19$, however using the adjacency graph we could still construct the colorings with a very low MLS cost. We did not compute MLS values of minimum linear clusterings for $k>19$

Similar plots are provided for $k=6$ and 12 in Fig. 17 and for $k=18$ and 24 in Fig. 18. They indicate that the mean of MLS stays similar for linear and k-medoids and increases with *k*. The divergence from linearity of k-medoids for normalized Hi-C does too, while that of non-normalized Hi-C stays close to 5.

## Conclusion and further work

Aside from problems that consider rearrangement length, little is known about weighted rearrangements [16–20]. In [9], we showed that with a simple cost function based on a partition of the adjacencies of one of the genomes into equivalence classes, one can choose—from the exponentially large set of shortest scenarios—a scenario that minimizes the number of moves acting across classes.

In this paper we showed that the genome rearrangement problem with an objective function based solely on the cost of DCJs is NP-Hard, even for a simple binary cost function. We gave a 3/2-approximation derived from bounds on the sizes of cycles in a cycle packing of the junction graph. We also presented an exact algorithm and found that an exact solution can be computed quickly between *D. melanogaster* and *D. yakuba*.

Our algorithms depend on a coloring of the adjacencies between syntenic blocks. We use MLS to study these colorings by clustering normalized and non-normalized Hi-C data from *D. melanogaster*. We find that our clusterings based on normalized data with the unsophisticated *k*-medoids technique give marginally lower MLS costs than clusterings that strictly preserve the linear ordering of the adjacencies. Both of those non-random clusterings give much lower MLS costs than randomized clusterings. Our rough linearity measure shows that, as *k*-medoid clusterings become more linear, the cost of the MLS decreases. A concerted effort towards the development of normalization and clustering is required to study these relationships in more detail.

Our model supports the coloring of a single genome only, yet this coloring may be capable of representing the 3D spatial structure between two genomes. Indeed, if spatial organization is somewhat conserved across large evolutionary distances, the chromatin conformation from a single genome could be informative for the inference of rearrangements over an entire scenario. Such conservation has been demonstrated in at least two cases. Recent results show that syntenic regions in mouse and human share a high degree of similarity in their higher order chromatin structure [21]. Further, syntenic blocks distant in the 1D sense in human, but adjacent in mouse, were observed in close 3D proximity in the human more often than expected; the authors concluded that there is a certain degree of conservation in spatial structure [8]. Ideally, adjacencies in these conserved regions would get the same color. Despite this large-scale conservation, however, further extension of the model to accommodate discrepancies in the Hi-C data between species is of future interest.

Our work opens the door to the development of more complex models of genome rearrangement with positional constraints, where local moves would be attributed nonzero cost. To this end we established a useful link between the weighted distance and the difference between minimum local parsimonious scenario and minimum local scenario in the "Towards a more general cost function" section. Preliminary experimental results indicate that the problem of finding a minimum cost scenario among those of almost minimum length would be of practical interest.
